# Gender differences in association between uric acid and all-cause mortality in patients with chronic heart failure

**DOI:** 10.1186/s12872-018-0989-8

**Published:** 2019-01-05

**Authors:** Viera Stubnova, Ingrid Os, Aud Høieggen, Marit D. Solbu, Morten Grundtvig, Arne S. Westheim, Dan Atar, Bård Waldum-Grevbo

**Affiliations:** 1 Finnmark Hospital Trust, Kirkenes, Norway; 2 Institute of Clinical Medicine, University of Oslo, Oslo, Norway; 3 Department of Nephrology, Oslo University Hospital, Ullevål, Oslo, Norway; 4 Section of Nephrology, University Hospital of North Norway, Tromsø, Norway; 5 Metabolic and Renal Research Group, UiT The Arctic University of Norway, Tromsø, Norway; 6 Department of Medicine, Innlandet Hospital Trust, Lillehammer, Norway; 7 Department of Cardiology, Oslo University Hospital, Ullevål, Oslo, Norway

**Keywords:** Uric acid, Heart failure, Gender, Kidney disease, All-cause mortality, Propensity score, Epidemiology

## Abstract

**Background:**

Elevated serum uric acid (SUA) is associated with poor prognosis in patients with cardiovascular disease, yet it is still not decided whether the role of SUA is causal or only reflects an underlying disease. The purpose of the study was to investigate if SUA was an independent predictor of 5-year all-cause mortality in a propensity score matched cohort of chronic heart failure (HF) outpatients. Furthermore, to assess whether gender or renal function modified the effect of SUA.

**Methods:**

Patients (*n* = 4684) from the Norwegian Heart Failure Registry with baseline SUA were included in the study. Individuals in the highest gender-specific SUA quartile were propensity score matched 1:1 with patients in the lowest three SUA quartiles. The propensity score matching procedure created 928 pairs of patients (73.4% males, mean age 71.4 ± 11.5 years) with comparable baseline characteristics. Kaplan Meier and Cox regression analyses were used to investigate the independent effect of SUA on all-cause mortality.

**Results:**

SUA in the highest quartile was an independent predictor of all-cause mortality in HF outpatients (hazard ratio (HR) 1.19, 95% confidence interval (CI) 1.03–1.37, *p*-value 0.021). Gender was found to interact the relationship between SUA and all-cause mortality (*p*-value for interaction 0.007). High SUA was an independent predictor of all-cause mortality in women (HR 1.65, 95% CI 1.24–2.20, *p*-value 0.001), but not in men (HR 1.06, 95% CI 0.89–1.25, *p*-value 0.527). Renal function did not influence the relationship between SUA and all-cause mortality (*p*-value for interaction 0.539).

**Conclusions:**

High SUA was independently associated with inferior 5-year survival in Norwegian HF outpatients. The finding was modified by gender and high SUA was only an independent predictor of 5-year all-cause mortality in women, not in men.

## Background

The relationship between elevated serum uric acid (SUA) and cardiovascular (CV) disease and mortality is well recognized [1, 2], yet it is still undecided whether the association reflects a causal inference or whether SUA is a risk marker reflecting the burden of the underlying disease.

SUA, the end product of purine metabolism in humans, is catalysed by xanthine oxidase (XO) and predominantly eliminated by the kidneys [3]. Renal function, gender, race, and medication may all influence SUA level [2]. In addition, genetic studies have uncovered variants in urate reabsorption and excretion transporters that are responsible for some variation in SUA level [4].

High SUA in heart failure (HF) may result from impaired oxidative metabolism causing accumulation of uric acid precursors and increased XO activation [5] as well as from decreased renal elimination as chronic kidney disease (CKD) is highly prevalent [6].

High SUA levels have been found to be related to incident HF [7–10] and to be associated with poor outcomes in HF patients [11–14]. An association between SUA and incident, prevalent and progressive CKD has also been detected [15–17] but the results concerning effect of SUA on mortality in CKD patients are inconsistent [18–21].

Cardiovascular risk factors and outcomes differ between men and women [22]. Gender differences are also apparent in HF patients, both with regard to aetiology, left ventricle ejection fraction (LVEF) and prognosis [23–26]. The association between SUA and CV disease outcomes appears to be more pronounced in women than in men [7, 27, 28] but the role of gender in the relationship between SUA and survival of HF patients is not yet clearly determined.

Reducing the effect of confounding is crucial when estimating associations in observational studies. Propensity score matching is a statistical method that accounts for confounding variables in a different manner than traditional multivariate Cox proportional hazards model and might be a superior method [29].

The aim of the current study was to examine whether SUA is an independent predictor of all-cause mortality in a propensity score matched cohort of Norwegian HF outpatients. Furthermore, we aimed to analyse if the effect of SUA on all-cause mortality is modified by gender or renal function.

## Methods

### The Norwegian heart failure registry

The Norwegian Heart Failure Registry has collected data on outpatients referred to HF clinics in Norwegian hospitals since 2000. By February 2012, a total of 6675 patients were enrolled by 25 HF clinics in different Norwegian regions that cover about half of Norway’s population. The participating HF clinics were run by cardiologists and specialized nurses. Patients were registered after they had been diagnosed with chronic HF of any aetiology following the guidelines of the European Society of Cardiology (ESC) [30, 31]. Three visits were recorded. At the time of the first visit (baseline), medical history, physical examination, echocardiography, New York Heart Association (NYHA) functional class, laboratory results, and the medical management of HF were recorded. The last adjustment visit was recorded at stable follow-up, after the multidisciplinary team had optimized the treatment and the patient had participated in an educational program. At the time of the third visit, arranged 6 months after the last adjustment visit, patient’s health condition was reassessed, as well as medication and laboratory results. Mortality data are retrieved yearly from Statistics Norway.

### Study population

A total of 4953 (74.2%) patients in the Norwegian Heart Failure Registry had available baseline measurements of SUA and were eligible for the study. The patients in each reporting hospital were grouped into gender specific SUA quartiles, as the participating hospitals used different laboratory assays for SUA analyses and the recommended reference range of SUA differs for women and men (women 18–49 years: 155–350 μmol/l, women over 50 years: 155–400 μmol/l, men: 230–480 μmol/l) [32]. Subjects from hospitals with less than 40 registered subjects were excluded to achieve proper stratification. Consequently, 4684 patients from 19 hospitals were stratified and included in the analyses. Finally, patients in each SUA quartile were merged together across hospitals and gender, comprising about 1180 subjects in each group.

### Definitions

Renal function was expressed as estimated glomerular filtration rate (eGFR) and calculated using the Chronic Kidney Disease Epidemiology Collaboration (CKD-EPI) equation [33]. Reduced renal function was defined as eGFR< 60 ml/min/1.73 m^2^.

Based on 2016 ESC Guidelines on HF [34], LVEF was defined as reduced at < 40% and as preserved at ≥50%.

Diagnosis of hypertension was based on information on antihypertensive treatment.

Daily doses of angiotensin-converting enzyme inhibitors (ACEi) were converted to enalapril equivalent doses (enalapril 20 mg = lisinopril 20 mg = ramipril 10 mg = captopril 100 mg), and then expressed as percent of enalapril target dose. Target dose of enalapril was defined as 20 mg per day. Daily doses of loop diuretics were converted to furosemide equivalent doses (furosemide 40 mg = bumetanide 1 mg). Daily doses of β-blockers were converted to metoprolol equivalent doses (metoprolol 200 mg = bisoprolol 10 mg = carvedilol 50 mg = atenolol 100 mg).

### Statistical analysis

Continuous variables were expressed as mean ± standard deviation and categorical variables as frequencies (percentage). Differences in continuous variables were compared by one-way analysis of variance and Student t-test as required. Similarly, differences in categorical variables were compared by χ^2^ test. The two-tailed significance level test was set to *p* < 0.05.

An individual propensity score, the likelihood of SUA being in the highest quartile, was obtained for each patient using a multivariate logistic regression model. Baseline variables found to be associated with SUA in the highest quartile (*p*-value < 0.10) and variables that could potentially confound the relationship between SUA and mortality were chosen as independent variables when calculating the propensity score. The following 16 covariates were entered in the model: gender, age, body mass index (BMI), smoking, diabetes mellitus, claudication and/or previous stroke, systolic blood pressure, NYHA functional class, use of renin-angiotensin-system (RAS)-blocking agents, β-blocker dose, diuretic dose, use of statin, eGFR, haemoglobin, serum sodium and serum potassium. Patients with SUA in the fourth quartile were then matched 1:1 to patients with SUA in quartiles 1–3 on the propensity score, using match tolerance of 0.05 with no replacement and preference to exact match.

Five-year survival curves were presented using Kaplan-Meier statistics. Univariate Cox proportional hazards model was used in the propensity score matched cohort and presented as hazard ratio (HR) and 95% confidential interval (95% CI). Due to the limited number of female patients, multivariable Cox proportional hazards model was used when evaluating the effect of SUA on all-cause mortality in the gender-stratified model. Baseline variables found to be associated with SUA in the highest quartile in women (*p*-value< 0.10) were included in the multivariate model: age, BMI, smoking, ischaemic heart disease, diabetes mellitus, hypertension, NYHA functional class, systolic blood pressure, LVEF, use of RAS-blocking agents, β-blocker dose, diuretic dose, eGFR, and serum sodium.

All statistical analyses were performed using IBM SPSS Statistics version 25 (IBM SPSS Statistics, New York, USA). Kaplan Meier survival curves were obtained using STATA/SE version 14.1 (StataCorp LP, Texas, USA).

## Results

### Baseline characteristics and propensity score matching

Baseline characteristics of the 4684 included HF outpatients are presented by SUA quartiles in Table 1. The mean age was 69.6 ± 12.2 years and 73.3% were males. Patients in higher SUA quartiles were more prone to be older, to have a history of diabetes and hypertension, more severe HF symptoms, higher BMI and worse renal function compared to patients in the lower SUA quartiles. They used higher doses of diuretics and β-blockers and were less likely to use RAS-blocking agents and acetylsalicylic acid. The median follow-up was 50 (interquartile range (IQR) 27, 78) months.

**Table 1:** Baseline characteristics of HF outpatients before and after propensity score matching, by SUA quartiles

	Quartiles of SUA in 4684 HF outpatients					1856 HF Outpatients after PSM		
	1 (*n* = 1187)	2 (*n* = 1169)	3 (*n* = 1154)	4 (*n* = 1174)	*P*-value	SUA Quartile 1–3 (*n* = 928)	SUA Quartile 4 (*n* = 928)	*P*-value
Se-uric acid, μmol/L	310.6 ± 51.5	405.4 ± 35.8	490.3 ± 36.5	635.2 ± 88.2	< 0.001	427.7 ± 80.3	633.3 ± 85.3	< 0.001
Se-uric acid, mg/dL	5.22 ± 0.87	6.82 ± 0.60	8.24 ± 0.61	10.68 ± 1.48		7.19 ± 1.35	10.65 ± 1.43	
Male gender, %	73.0	73.7	74.5	71.9	0.527	73.5	73.3	0.916
Age, years	68.0 ± 12.8	68.8 ± 11.9	69.5 ± 12.1	71.9 ± 11.1	< 0.001	71.3 ± 11.3	71.4 ± 11.7	0.770
Body mass index, kg/m^2^	25.3 ± 4.7	26.2 ± 5.0	27.0 ± 5.3	26.9 ± 5.3	< 0.001	26.5 ± 5.2	26.6 ± 5.1	0.683
Smoking, %	18.5	15.9	13.8	13.0	0.001	14.3	13.8	0.738
Medical history								
Diabetes mellitus, %	15.9	18.7	19.8	24.0	< 0.001	20.8	21.6	0.691
Ischaemic heart disease, %	55.1	54.5	56.1	58.1	0.334	57.8	57.5	0.919
Hypertension, %	22.9	32.8	33.7	38.8	< 0.001	36.8	38.7	0.395
Claudication/stroke, %	13.6	14.8	15.2	17.2	0.106	17.6	17.1	0.806
PCI/CABG, %	37.2	39.8	37.7	37.7	0.575	38.7	38.6	0.968
Reduced renal function, %	21.6	31.9	47.4	71.9	< 0.001	67.1	68.5	0.518
Physical findings								
Heart rate, beats/min	71.8 ± 14.3	71.9 ± 14.4	73.0 ± 15.5	73.6 ± 15.5	0.008	74.5 ± 16.1	73.7 ± 15.3	0.265
SBP, mmHg	127.9 ± 22.2	128.1 ± 22.7	127.0 ± 21.7	123.4 ± 22.5	< 0.001	125.3 ± 22.0	124.4 ± 22.4	0.398
LVEF, %	33.4 ± 11.1	32.7 ± 11.2	32.4 ± 11.6	32.4 ± 12.5	0.131	32.3 ± 11.7	32.2 ± 12.7	0.956
LVEF groups					0.171			0.557
LVEF< 40%	72.2	74.6	73.2	74.9		74.3	75.6	
40% ≤ LVEF< 50%	18.6	16.3	18.1	14.6		16.0	14.1	
LVEF≥50%	9.2	9.1	8.7	10.6		9.8	10.3	
NYHA Class					< 0.001			0.548
I + II, %	58.4	52.2	47.8	37.6		39.8	36.9	
III + IV, %	41.7	47.9	52.3	62.4		60.3	63.0	
Medication								
RAS-blocking agent use, %	89.0	90.8	90.1	87.2	0.027	88.8	88.1	0.663
ACEi dose/day, % of target dose	48.1 ± 36.4	53.1 ± 37.8	54.9 ± 38.0	52.9 ± 41.8	0.001	51.6 ± 38.3	53.1 ± 42.2	0.486
ARB use, %	14.2	14.7	16.8	17.4	0.089	16.6	17.0	0.804
β-blocker dose/day, mg	61.1 ± 58.2	74.2 ± 67.4	72.3 ± 61.8	77.7 ± 66.7	< 0.001	76.8 ± 66.1	75.2 ± 65.6	0.605
Loop diuretics dose/day, mg	34.4 ± 43.9	47.6 ± 53.6	62.9 ± 48.5	87.5 ± 72.5	< 0.001	72.2 ± 70.5	83.4 ± 70.4	0.001
Calcium channel blocker use, %	7.4	8.2	8.3	8.4	0.785	8.9	7.3	0.225
Acetylsalicylic acid use, %	51.3	47.9	45.5	43.4	0.001	44.5	43.4	0.644
Statin use, %	56.0	56.1	54.3	51.8	0.124	51.9	51.6	0.889
Laboratory values								
eGFR, ml/min/1.73 m^2^	75.3 ± 20.4	69.2 ± 20.5	62.6 ± 21.0	50.9 ± 20.7	< 0.001	54.1 ± 19.8	52.9 ± 20.8	0.205
Haemoglobin, g/100 mL	13.79 ± 1.57	14.00 ± 1.65	13.89 ± 1.72	13.70 ± 1.89	< 0.001	13.78 ± 1.78	13.75 ± 1.85	0.700
Se-potassium, mmol/L	4.38 ± 0.41	4.41 ± 0.43	4.38 ± 0.49	4.41 ± 0.52	0.136	4.43 ± 0.50	4.40 ± 0.50	0.280
Se-sodium, mmol/L	139.7 ± 3.3	140.0 ± 3.1	140.0 ± 3.2	139.7 ± 3.4	0.024	139.8 ± 3.3	139.7 ± 3.3	0.530
Se-cholesterol, mmol/L	4.65 ± 1.23	4.71 ± 1.22	4.79 ± 1.31	4.72 ± 1.33	0.097	4.73 ± 1.29	4.75 ± 1.31	0.745

Values are expressed as mean ± SD or percent. ACEi dose/day, percent of daily enalapril equivalent target dose; ARB, angiotensin receptor blocker; β-blocker dose/day, daily metoprolol equivalent dose; eGFR, estimated glomerular filtration rate; HF, heart failure; LVEF, left ventricular ejection fraction; NYHA, New York Heart Association; PCI/CABG, percutaneous coronary intervention and/or coronary artery bypass graft; PSM, propensity score matching; RAS-blocking agent, renin-angiotensin system blocking agent; SBP, systolic blood pressure; SUA, serum uric acid

**Fig. 1:**
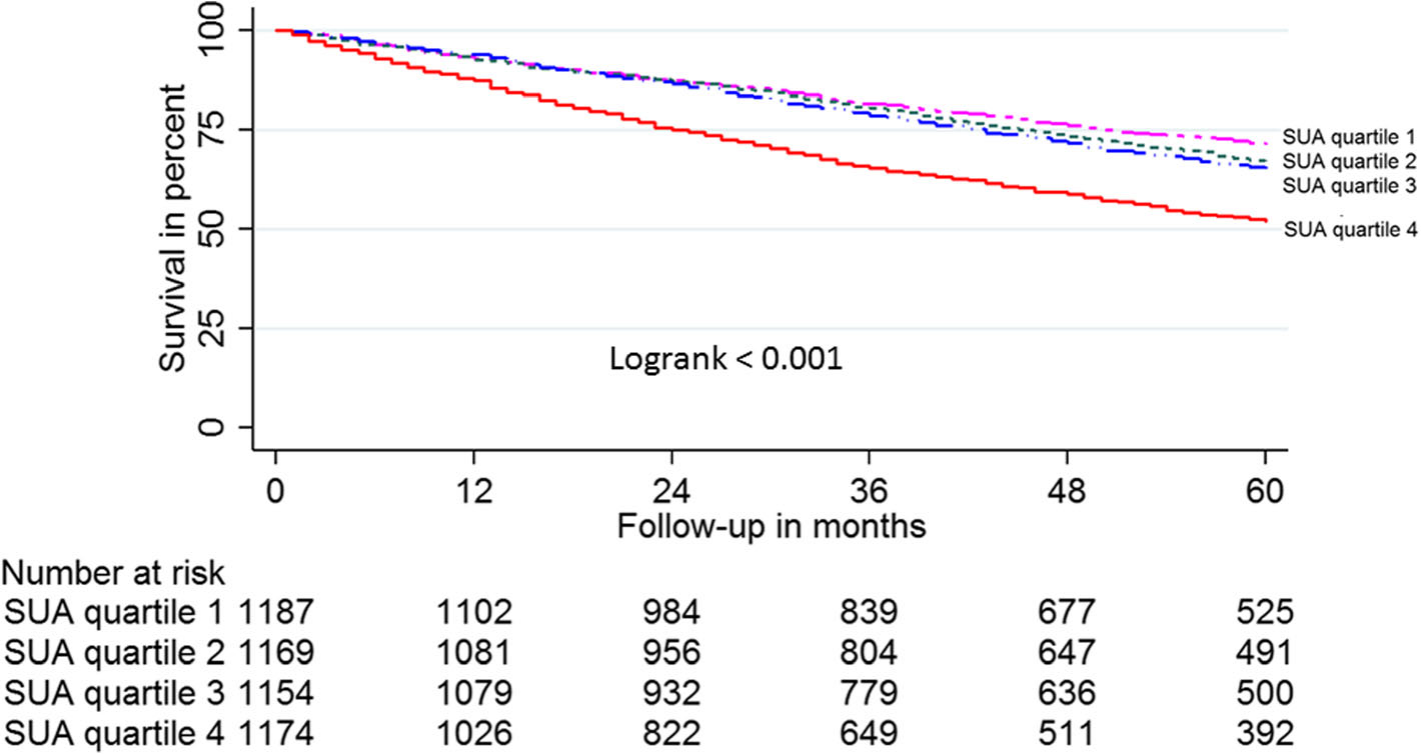
Kaplan-Meier survival plot of 4684 HF outpatients according to SUA quartile

Kaplan-Meier survival curves for SUA in quartiles 1–3 were almost superimposable and all-cause mortality for individuals with SUA in quartile 4 was significantly greater than for those with SUA in quartiles 1–3 (log-rank < 0.001, Fig. 1). Individuals with SUA in the lowest three quartiles were therefore all selected to be potential controls in the propensity matched model. A total of 928 subjects with SUA in quartile 4 were matched 1:1 by propensity score to subjects with SUA in quartiles 1–3. Baseline characteristics of the 1856 propensity score matched subjects were well-balanced (Table 1).

### Survival analyses and outcomes based on SUA level

**Fig. 2:**
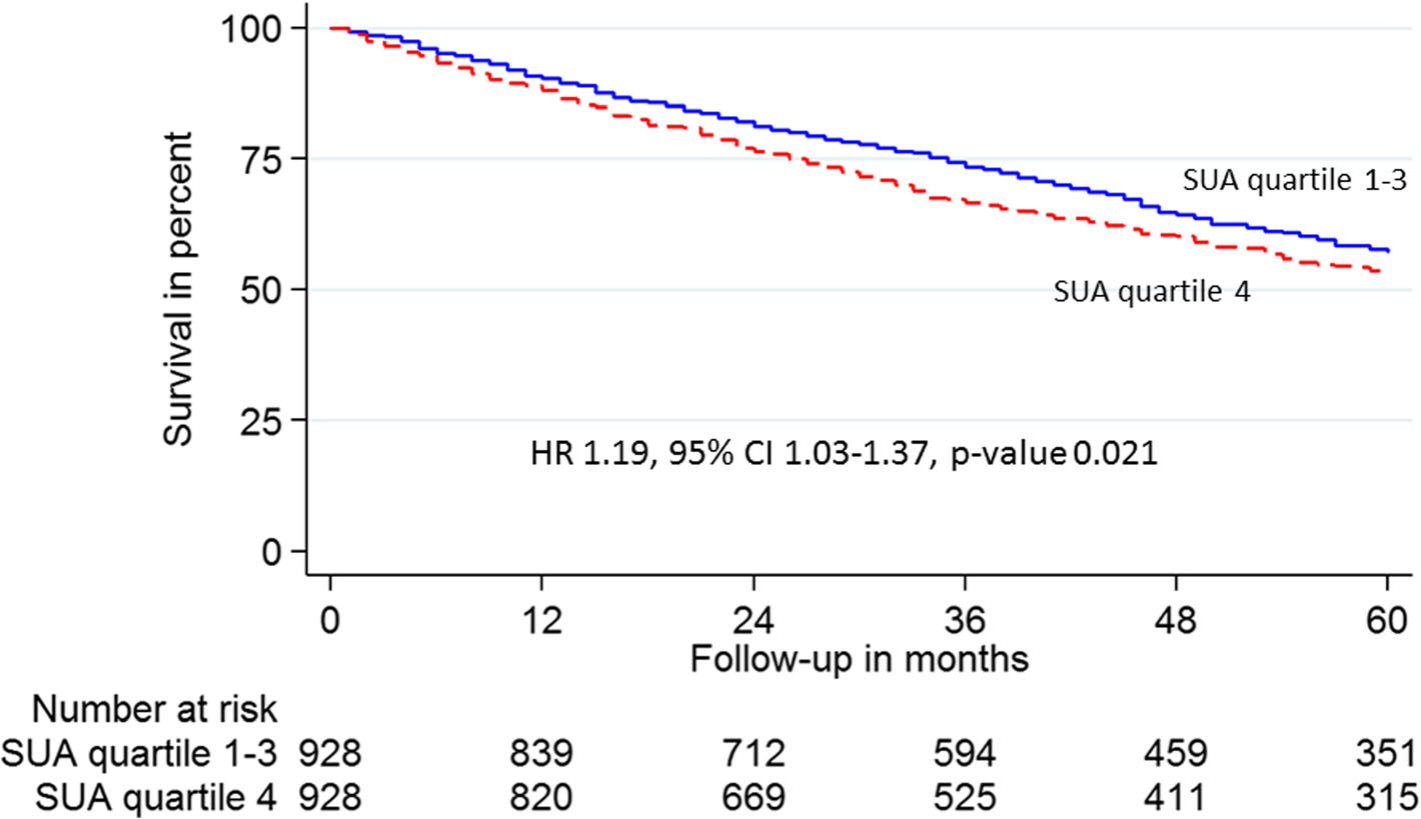
Kaplan-Meier survival plot of 1856 HF outpatients propensity score matched by SUA in quartile 4

SUA in the highest quartile was an independent predictor of all-cause mortality in HF outpatients (HR 1.19, 95% CI 1.03–1.37, *p*-value 0.021, Fig. 2).

**Fig. 3:**
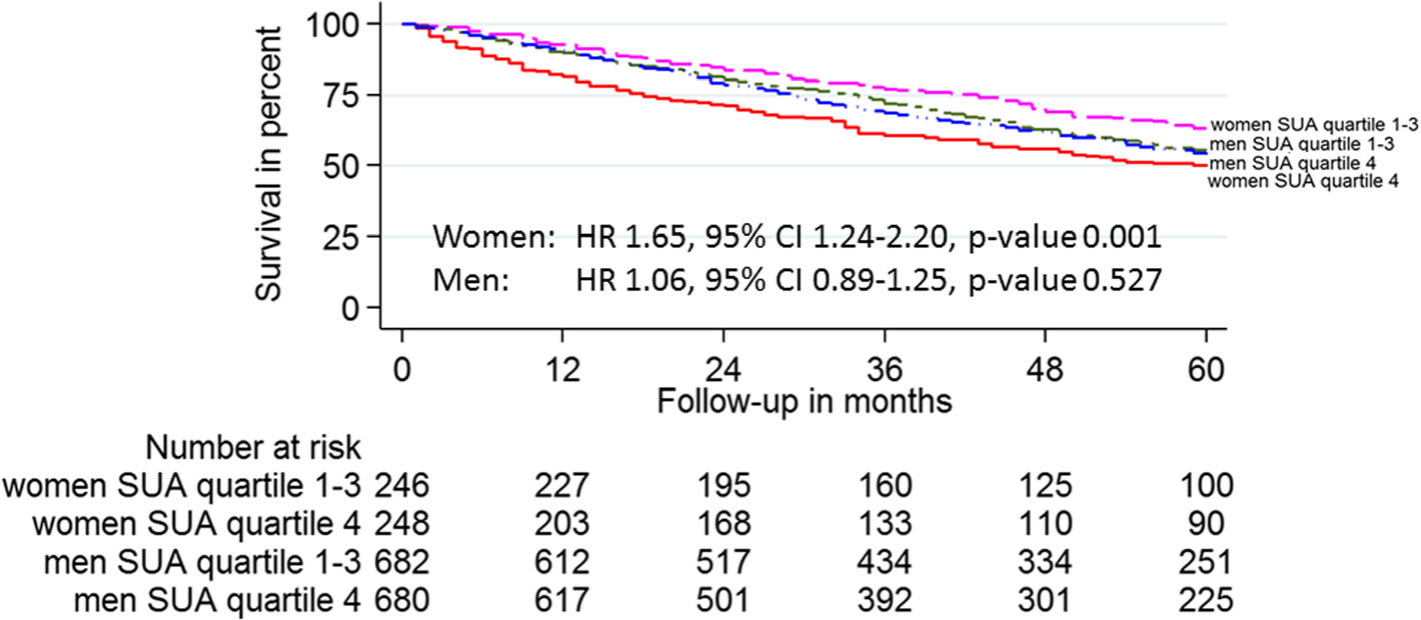
Kaplan-Meier survival plot of propensity score matched HF outpatients according to gender and SUA quartile

Gender was found to interact the relationship between SUA and all-cause mortality in the propensity matched model (*p*-value for interaction 0.007). Differences in the survival of HF outpatients depending on gender and SUA quartile are depicted in Kaplan-Meier survival curves in Fig. 3. High SUA was an independent predictor of all-cause mortality in women (HR 1.65, 95% CI 1.24–2.20, *p*-value 0.001) but not in men (HR 1.06, 95% CI 0.89–1.25, *p*-value 0.527). Renal function did not interact the relationship between SUA and all-cause mortality (*p*-value for interaction 0.539).

Women and men with SUA in the highest quartile differed both in age, comorbidity, medication, and physical and laboratory findings from those with lower SUA (Table 2). The number of female patients was limited and a gender-stratified propensity matched model was not possible. Subsequently, gender specific multivariate Cox proportional hazard model analyses in the subgroups of 1251 female and 3433 male HF outpatients were performed to further explore gender differences in the prognostic value of SUA on survival. In the subgroup of female HF outpatients, SUA in the highest quartile was confirmed to be an independent predictor of all-cause mortality (HR 1.51, 95% CI 1.13–2.02, *p*-value 0.005). On the contrary, SUA did not independently predict all-cause mortality in the subgroup of male HF outpatients (HR 1.10, 95% CI 0.94–1.30, *p*-value 0.249).

**Table 2:** Gender-specific baseline characteristics of HF outpatients, by SUA quartiles

	All (*n* = 4684)			Women (*n* = 1251)			Men (*n* = 3443)		
	Women (*n* = 1251)	Men (*n* = 3433)	*P*-value	SUA Quartile 1–3 (*n* = 921)	SUA Quartile 4 (*n* = 330)	*P*-value	SUA Quartile 1–3 (*n* = 2589)	SUA Quartile 4 (*n* = 844)	*P*-value
Se-uric acid, μmol/L	435.2 ± 143.9	468.9 ± 127.1	< 0.001	367.4 ± 86.1	624.1 ± 96.6	< 0.001	413.3 ± 80.7	639.6 ± 84.3	< 0.001
Se-uric acid, mg/dL	7.32 ± 2.42	7.88 ± 2.14		6.18 ± 1.45	10.49 ± 1.62		6.95 ± 1.36	10.75 ± 1.42	
Age, years	72.1 ± 12.1	68.6 ± 12.1	< 0.001	70.7 ± 12.3	76.2 ± 10.4	< 0.001	68.1 ± 12.2	70.3 ± 11.6	< 0.001
Body mass index, kg/m^2^	25.6 ± 5.6	26.6 ± 4.9	< 0.001	25.4 ± 5.6	26.1 ± 5.4	0.054	26.4 ± 4.9	27.1 ± 5.2	0.001
Smoking, %	12.6	16.3	0.002	14.3	7.9	0.002	16.8	15.1	0.247
Medical history									
Diabetes mellitus, %	18.7	19.9	0.352	16.6	24.6	0.001	18.7	23.7	0.001
Ischaemic heart disease, %	46.0	59.6	< 0.001	44.2	50.8	0.045	59.1	61.1	0.323
Hypertension, %	37.1	30.2	< 0.001	33.5	47.1	< 0.001	28.4	35.6	< 0.001
Claudication/stroke, %	13.6	15.8	0.071	13.4	14.1	0.777	14.9	18.4	0.016
PCI/CABG, %	26.1	42.5	< 0.001	25.9	26.7	0.774	42.6	42.0	0.746
Reduced renal function, %	51.3	40.1	< 0.001	39.7	83.6	< 0.001	31.3	67.3	< 0.001
Physical findings									
Heart rate, beats/min	73.5 ± 14.3	72.2 ± 15.2	0.008	73.1 ± 14.5	74.6 ± 13.7	0.105	71.9 ± 14.8	73.1 ± 16.1	0.044
SBP, mmHg	128.2 ± 23.1	126.0 ± 22.0	0.004	128.9 ± 23.2	126.3 ± 22.8	0.079	127.2 ± 21.8	122.3 ± 22.4	< 0.001
LVEF, %	35.8 ± 13.3	31.7 ± 10.8	< 0.001	35.3 ± 12.5	37.1 ± 15.3	0.092	32.0 ± 10.7	30.8 ± 10.9	0.007
LVEF groups			< 0.001			0.023			0.031
LVEF< 40%	64.6	76.8		66.1	59.8		75.8	80.0	
40%≤LVEF<50%	19.5	16.0		19.7	18.9		17.0	13.1	
LVEF≥50%	15.9	7.2		14.1	21.2		7.2	6.9	
NYHA Class			0.001			< 0.001			< 0.001
I + II, %	44.1	50.8		50.0	27.5		53.8	41.5	
III + IV, %	55.9	49.2		50.0	72.5		46.2	58.4	
Medication									
RAS-blocking agent use, %	87.3	90.0	0.008	88.7	83.3	0.012	90.4	88.7	0.159
ACEi dose/day, % of target dose	49.3 ± 40.0	53.2 ± 38.0	0.005	49.3 ± 36.9	49.4 ± 47.8	0.975	52.9 ± 37.7	54.2 ± 39.2	0.452
ARB use, %	17.4	15.2	0.060	16.8	19.1	0.353	14.7	16.7	0.144
β-blocker dose/day, mg	67.6 ± 62.2	72.6 ± 64.5	0.017	65.6 ± 60.0	73.1 ± 14.5	0.059	70.4 ± 63.7	79–4 ± 66.2	< 0.001
Loop diuretics dose/day, mg	86.7	85.1	0.149	44.2 ± 45.6	90.4 ± 83.1	< 0.001	49.2 ± 51.5	86.4 ± 67.9	< 0.001
Calcium channel blocker use, %	9.2	7.6	0.080	8.6	10.9	0.205	7.7	7.4	0.727
Acetylsalicylic acid use, %	44.4	48.0	0.027	45.3	41.8	0.278	49.3	44.0	0.007
Statin use, %	46.7	57.4	< 0.001	47.7	43.9	0.245	58.2	54.9	0.086
Laboratory values									
eGFR, ml/min/1.73 m^2^	60.3 ± 22.7	66.1 ± 22.3	< 0.001	66.0 ± 21.5	44.6 ± 18.1	< 0.001	70.2 ± 21.1	53.4 ± 21.1	< 0.001
Haemoglobin, g/100 mL	13.31 ± 1.53	14.04 ± 1.74	< 0.001	13.32 ± 1.43	13.21 ± 1.77	0.202	14.09 ± 1.68	13.90 ± 1.90	0.010
Se-potassium, mmol/L	4.32 ± 0.48	4.42 ± 0.46	< 0.001	4.32 ± 0.45	4.32 ± 0.53	0.950	4.41 ± 0.44	4.45 ± 0.51	0.055
Se-sodium, mmol/L	139.4 ± 3.1	140.0 ± 3.1	< 0.001	139.6 ± 3.5	139.1 ± 3.5	0.047	140.0 ± 3.1	139.9 ± 3.1	0.331
Se-cholesterol, mmol/L	5.08 ± 1.34	4.58 ± 1.22	< 0.001	5.06 ± 1.33	5.11 ± 1.38	0.588	4.59 ± 1.20	4.56 ± 1.28	0.561

Values are expressed as mean ± SD or percent. ACEi dose/day, percent of daily enalapril equivalent target dose; ARB, angiotensin receptor blocker; β-blocker dose/day, daily metoprolol equivalent dose; eGFR, estimated glomerular filtration rate; HF, heart failure; LVEF, left ventricular ejection fraction; NYHA, New York Heart Association; PCI/CABG, percutaneous coronary intervention and/or coronary artery bypass graft; RAS-blocking agent, renin-angiotensin system blocking agent; SBP, systolic blood pressure; SUA serum uric acid

## Discussion

The current study demonstrates that high level of SUA was an independent predictor of 5-year all-cause mortality in patients with chronic HF. The finding was gender specific and only found in women. To our knowledge, this is the first propensity score matched study to report the gender modifying effect on the relationship between SUA and all-cause mortality in chronic HF. The predictive value of SUA on mortality was not modified by renal function.

Other studies have found an association between high levels of SUA and poor outcome in chronic HF patients [13, 21, 35–37], still the causal relationship is considered undecided. We report SUA in the fourth quartile to be an independent predictor of all-cause mortality selectively in women, both in the propensity score matched model and multivariate Cox regression model.

Gender differences in the effect of SUA on outcomes have been reported previously in patients with CV disease. In hypertensive patients with left ventricular hypertrophy, the association between SUA and CV events was reported to be stronger in women than in men [28]. A study of patients with acute coronary syndrome showed that SUA was predictive of CV events in women but not in men [38]. Similarly, in a population based survey, SUA was found to be an independent predictor of mortality in women only [27]. Our results now expand the evidence for gender differences in the effect of SUA also to be valid in HF outpatients.

In most previous studies assessing differences in survival between men and women with HF, women have been reported to have better survival than men [25, 39–44]. Sex hormones affect myocardial calcium handling, nitric oxide, glucose and fatty metabolism as well as cardiac fibrosis, and may participate in the mechanisms for differences between female and male failing hearts [26]. SUA is a potent antioxidant but at the same time, SUA and XO lead to reduced nitric oxide bioavailability, ensuing endothelial dysfunction, inflammation and vasoconstriction [45]. Menopause has been found to be associated with increasing SUA, possibly due to altered effect of oestrogen on renal tubular handling of uric acid [46]. We did not have information on menopausal status in female HF outpatients in the current study, but the mean age of 72.1 ± 12.1 years implies that the great majority were postmenopausal. Our study revealed distinct differences between women and men with SUA in quartile 4 with regard to age, type, symptoms and treatment of HF, as well as comorbidity and renal function. Still, both the propensity score matched model and multivariate Cox regression model identified SUA in the highest quartile to be a predictor of all-cause mortality in women independently of the above mentioned confounding variables. The mechanisms for the deteriorating effect of high SUA on survival selectively in postmenopausal women need to be further explored, yet our findings may imply SUA being a future treatment target in female HF patients. Urate-lowering therapy is currently not recommended in asymptomatic hyperuricemia due to limited benefit-risk data in non-gout diseases [47]. Nevertheless, XO-inhibiting therapy has been shown to have beneficial effects in some patient groups [48]. In HF patients with hyperuricemia, XO-inhibition did not improve survival, but it is noteworthy that the study was not gender stratified and only of 24-week duration [49].

Renal function did not modify the effect of SUA on all-cause mortality in the present study. This corroborates the observation by Anker et al. [13] who also found SUA to be a predictor of poor outcome in HF independent of renal function, while Filippatos et al. [21] found SUA to be associated with poor outcome only in HF patients without CKD. Studies exploring SUA impact in patients with CKD show inconsistent results [18–20].

Some limitations of our study need to be considered. Because of various laboratory assays for SUA analyses in the reporting hospitals, we grouped patients in each hospital into gender-specific SUA quartiles. Small groups may cause a systematic error and therefore we did not include patients from hospitals with less than 40 registered individuals. On the other hand, we might have introduced a selection bias by excluding some hospitals. Patients in each SUA quartile were merged together across hospitals and gender, eventually leading to some overlapping SUA values in the four quartiles.

We used both propensity score method and multivariate Cox regression method to reduce the bias by confounding. Propensity score matching is an increasingly used method that mimics some characteristics of randomized control trials (RCT) and makes it possible to directly compare outcomes in the two studied groups [29]. We used propensity score matching when assessing the impact of high SUA on survival in all HF outpatients. Propensity score for having SUA in the highest quartile was estimated based on 16 measured baseline variables. Two groups of patients were established based on propensity score, differing in the presence or absence of SUA in the fourth quartile and, similarly to RCTs, we could then directly compare survival in the groups. Distribution of baseline characteristic in the propensity matched groups was well-balanced except for daily doses of diuretics. However, the difference was minor and is unlikely to explain the disparity in survival. Furthermore, the large size of the study population and the high number of variables used for estimation of propensity score and the fact that nearly 80% of patients with SUA in the highest quartile were propensity score matched should ensure reliability of our results. In the gender stratified analyses, we used multivariate Cox proportional hazard model to correct for the confounding variables as propensity score matching would lead to small size of the examined groups and thus could possibly introduce a selection bias. Yet, neither propensity score matching nor multivariate Cox proportional hazards method can correct for unmeasured confounding variables.

The current study is observational and therefore restricted to the existing data in the Norwegian Heart Failure Registry. We could not influence selection of the collected variables. Information on alcohol consumption, losartan use, hormone replacement therapy, the use of SUA lowering drugs, thyroid function, and triglycerides level could have added valuable information. At the same time, the observational nature of this study is among its strengths as the included patients represent a relatively unselected population in contrast to highly selected subjects in RCTs.

## Conclusions

SUA in the highest quartile was independently associated with inferior 5-year survival in Norwegian HF outpatients. The finding was modified by gender and high SUA was only an independent predictor of 5-year all-cause mortality in women but not in men. Our findings indicate that SUA might be a therapeutic target selectively in female HF patients. Renal function did not modify the effect of SUA on all-cause mortality.

## Abbreviations

ACEi: Angiotensin-converting enzyme inhibitors
ARB: Angiotensin II receptor blocker
BMI: Body mass index
CI: Confidence interval
CKD: Chronic kidney disease
CKD-EPI: Chronic Kidney Disease Epidemiology Collaboration
CV: Cardiovascular
eGFR: Estimated glomerular filtration rate
ESC: European Society of Cardiology
HF: Heart failure
HR: Hazard ratio
IQR: Interquartile range
LVEF: Left ventricle ejection fraction
NYHA: New York Heart Association
PCI/CABG: Percutaneous coronary intervention and/or coronary artery bypass graft
RAS: Renin-angiotensin system
SUA: Serum uric acid
XO: Xanthine oxidase
